# A network approach to rank countries chasing sustainable development

**DOI:** 10.1038/s41598-021-94858-2

**Published:** 2021-07-29

**Authors:** Carla Sciarra, Guido Chiarotti, Luca Ridolfi, Francesco Laio

**Affiliations:** DIATI, Politecnico di Torino, Corso Duca degli Abruzzi 24, 10129 Turin, Italy

**Keywords:** Complex networks, Sustainability

## Abstract

In 2015, the United Nations established the Agenda 2030 for sustainable development, addressing the major challenges the world faces and introducing the 17 Sustainable Development Goals (SDGs). How are countries performing in their challenge toward sustainable development? We address this question by treating countries and Goals as a complex bipartite network. While network science has been used to unveil the interconnections among the Goals, it has been poorly exploited to rank countries for their achievements. In this work, we show that the network representation of the countries-SDGs relations as a bipartite system allows one to recover aggregate scores of countries’ capacity to cope with SDGs as the solutions of a network’s centrality exercise. While the Goals are all equally important by definition, interesting differences self-emerge when non-standard centrality metrics, borrowed from economic complexity, are adopted. Innovation and Climate Action stand as contrasting Goals to be accomplished, with countries facing the well-known trade-offs between economic and environmental issues even in addressing the Agenda. In conclusion, the complexity of countries’ paths toward sustainable development cannot be fully understood by resorting to a single, multipurpose ranking indicator, while multi-variable analyses shed new light on the present and future of sustainable development.

## Introduction

Universality, integration, and inclusion: these are the principles and cornerstones upon which the United Nations (UN) have constructed, in 2015, the Agenda 2030 for sustainable development^1,2^. The Agenda, ratified by 193 countries, addresses through sustainable development the major challenges the world faces, such as environmental problems, climate change, economic growth, water, food and financial security, poverty and inequalities^3–5^; these also recently exacerbated by the Sars-CoV-2 pandemic^6,7^. The world is not new to the request of ‘a global agenda for change’. Back in 1987, the report “Our Common Future” already introduced the key idea of a common action plan to address economic growth in equilibrium with the people and environment, thus preserving our world to meet human needs for today’s and future generations^8^. The beginning of the XXI century marked a shift in the way countries started being actively engaged in the implementation of sustainable development, with the establishment of the Agenda 2015, allowing the joint forces of UN and governments to achieve significant milestones in poverty and inequalities reduction, as well as in improved water access^9,10^. In light of these achievements, and also of the limitations and gaps of such experience, the Agenda 2030 inherits and enlarges the views and objectives of the Agenda 2015^10^. In practical terms, today’s Agenda addresses a more equal, just, and sustainable future by introducing the 17 Sustainable Development GoalsSDGs^1^. The 17 Goals are constructed upon five pillars: people, prosperity, planet, peace and justice, and partnership; and connections and spillover effects among the Goals are unavoidably present^11–20^. In line with the Charter of the United Nations, the Sustainable Development Goals have no pyramidal structure, and there is no Goal prioritised with respect to the others, thus advocating for equal efforts in the designing of appropriate policies to meet these goals (Art. 40 of the Agenda)^1^. In fact, each Goal targets the implementation of policies, totalling 169 targets across the 17 Goals^18^. Targets also mark the need for data and measurements of the status of countries with respect to the achievement of the Goals. Countries ratifying the Agenda are encouraged to pursue sustainable development by defining national strategies with a global vision of their actions^1,2^, thus contributing to the common action plan necessary to foster change^1,17,21^ and embracing the cornerstones of the Agenda. Nevertheless, the Agenda is not a legal condition, and governments maintain sovereignty in choosing the most appropriate strategy to be placed in the field^1^. Moreover, on the one hand, countries exhibit remarkable heterogeneity in the challenges they have to face^1,6^; on the other hand, the interconnections among SDGs and their targets, also define trade-offs and synergies within different sectors of development^12,22^, which are enhanced by the strategies each country implements^23,24^. These factors unavoidably create different responses at the country level^4,5,25,26^.

It is clear then that the ensemble of countries and Goals within the Agenda 2030 is a complex system of its own^27^ (i.e., characterised by non-trivial and non-random interactions among many entities^28^), which requires proper mathematical approaches to monitor the status of countries, and able to account for their heterogeneity and the interconnections across the Goals. Indeed, such interconnections among the Goals and, no less, the synergies and trade-offs among development sectors, can be unveiled thanks to the use of complex network theory (see, e.g., Le Blanc^23^ and Guerrero et al.^18^). At the same time, within the development topic, the strategy of indexing is often used to rank countries for their performances, thus making the creation of aggregate scores necessary^29,30^ (notable examples are the Human Development Index^31^ and the Multidimensional Poverty Index^32^), and the Agenda 2030 makes no exception. To create aggregate scores of performances entails mathematically valuing each Goal’s contribution to the overall countries’ output, according to which compute a final score. In the construction of aggregate indices, many options can be pursued to weight these contributions^33,34^. A possible strategy would be to mathematically implement the egalitarian principle of the Agenda (i.e., all Goals must be of equal importance), thus entailing assigning the same weights of SDGs (see, e.g., the SDG Index by Sachs et al.^26,35,36^ and its applications at sub-national level^37^); nevertheless, other suitable strategies may exist (see, e.g., the Integrated Sustainable Development Index by Biggeri et al.^25^).

So far, the complex network analysis of the SDGs system and the creation of aggregate scores have been treated in parallel, without relevant overlaps. Instead, we argue that the combination of data and network science may help in disentangling the dynamics of development and defining data-driven weights to create more refined and comprehensive aggregate scores. In this work, we propose to tackle the definition of rankings of countries by promoting the use of the hidden bipartite network structure of the system defined by countries and Goals performances to highlight and unravel the intrinsic complexity of this system. Such representation of the Agenda 2030 allows one to use network methodologies to provide data-driven solutions to the problem of indexing of countries and weighting of the Goals.

## Results

### The complex network representation of countries and Sustainable Development Goals

As established by the United Nations^11^, progresses in the Sustainable Development Goals (and so, targets) are estimated using a set of indicators providing quantitative information about countries performances; each indicator measures the attainment of certain targets across the 17 SDGs. Let $I_{cgk}$ be the *k*-th value of the indicator *I* within Goal *g* recorded in country *c*. For the sake of comparison across indicators and Goals, most applications consider the $I_{cgk}$ values to be normalised according to least and optimal indicator values, resulting in a percentage of achievement of the indicator ranging from 0 to 100^25,36,38^ (see “Materials and methods” section). Moreover, per each country *c*, one single value of achievement *P* in each Goal *g* is obtained as the average of the recorded and available values of the indicator $I_{cgk}$ within the Goal. Namely,

1 $$\begin{aligned} P_{cg} = \frac{1}{N_{cg}}\sum _{k=1}^{N_{cg}} I_{cgk}, \end{aligned}$$

where $N_{cg}$ is the number of indicators in Goal *g* for country *c* (see “Materials and methods” section). An aggregate score $S_c$ of the countries’ status can be generally defined as a weighted sum of the Goal-specific performances

2 $$\begin{aligned} S_c \propto \sum _g P_{cg} \cdot w_g, \end{aligned}$$

where $w_g$ are the Goal-specific weights and the proportionality symbol considers the presence of any possible scaling factor.

**Figure 1:**
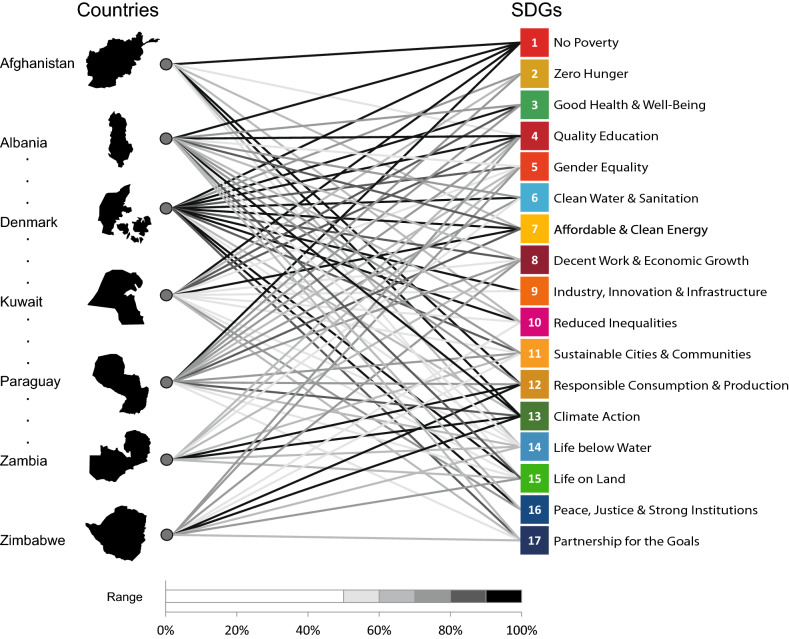
**The bipartite network of countries and Goals. **Qualitative representation of the bipartite network constituted by countries and Goals. On the left, we list seven of the countries that can be found by browsing the 2020 Dashboard, sorted in alphabetical order^26^ (the first and last two countries and the ones found at first, second and third quarter of the list). On the right, the 17 SDGs are reported. For each country, we connect the SDGs via the performance values $P_{cg}$ in each Goal, according to the 2020 Dashboard data^26^. The values $P_{cg}$ are intended to be readable as a percentage of achievement of the Goals. We have classed these values in ranges of $10\%$ of performances and colour-coded, in greyscale, accordingly: the darker the links, the better the performances of the country within the Goals. Countries’ performances smaller than $50\%$ have been left blank. The figure has been generated using Matlab R2019b, [https://www.mathworks.com/products/matlab.html], and PowerPoint 2016.

Within this framework, our aim is to cast the computation of aggregate scores of SDGs for countries through the use of network science to unveil and exploit the complex structure of the Agenda. Let us consider the values $P_{cg}$ as the starting point for our reasoning. We consider these values to be structured as a matrix $\mathbf {P}$ with *C* rows, i.e., the number of countries in the analysis, and 17 columns, as many as the Goals. Seen through network science lenses, the matrix $\mathbf {P}$ reveals the presence of a bipartite system in which countries and Goals are connected via recorded performances. In network theory, the matrix $\mathbf {P}$ describing these links is denominated as incidence matrix^39^. We consider the network structure of countries and Goals emerging from the data taken from the latest SDG Index and Dashboard, referring to the year 2020^26^ (see “Materials and methods” section), as exemplified in Fig. 1.

The bipartite network representation offers the chance to borrow mathematical tools of network’s centrality to define the importance of the nodes in the system and rank them accordingly^39^. Bipartite networks are characterised by the existence of two different sets of nodes, as in this case countries and Goals, and one centrality score can be computed for each set. The simplest measure of centrality, the nodes’ degree *k*, assumes the importance of the node to be described by the number and strength of its connections^39^. This provides the value $k_c = \sum _{g=1}^{17} P_{cg}$ for countries^39^, thus implicitly setting $w_g=1$ for all 17 Goals in the computation of the score $S_c$ in Eq. (2). (A mirror metric is defined for the SDGs; namely, the quantity $k_g=\sum _c P_{cg}$ defines the degree of a Goal *g*.) Notice that, in this countries-SDGs network, the link $P_{cg}$ between the nodes describes the existence of a connection between a country and a Goal but also the magnitude of this connection, represented by the recorded performance of the country in that SDG (as plot in Fig. 1). Therefore, according to the degree, $k_c$, countries with a higher percentage of achievement across SDGs have better chances of being central, so they are higher in ranking position, no matter the Goal. This rationale reflects the egalitarian principle of the Agenda, for which all SDGs have equal importance in being achieved^1^. We recall that, in light of this principle, the SDG Index by Sachs et al.^26^ is defined as^36^

$$\begin{aligned} SDG~Index = \frac{1}{17} \sum _{g=1}^{17} P_{cg}, \end{aligned}$$

and, one easily recognizes that the SDG Index corresponds to the degree centrality of countries ($k_c = \sum _g P_{cg}$) scaled by a factor 17.

The degree only measures the local information of nodes’ connections, and so it does not depict the global structure of the network (for further details see, e.g.,^40,41^). Therefore, although in line with the principle of the equal importance of SDGs, to rank countries with equal Goal weights entails not accounting for the complex interconnections in sustainable development we aforementioned.

**Figure 2:**
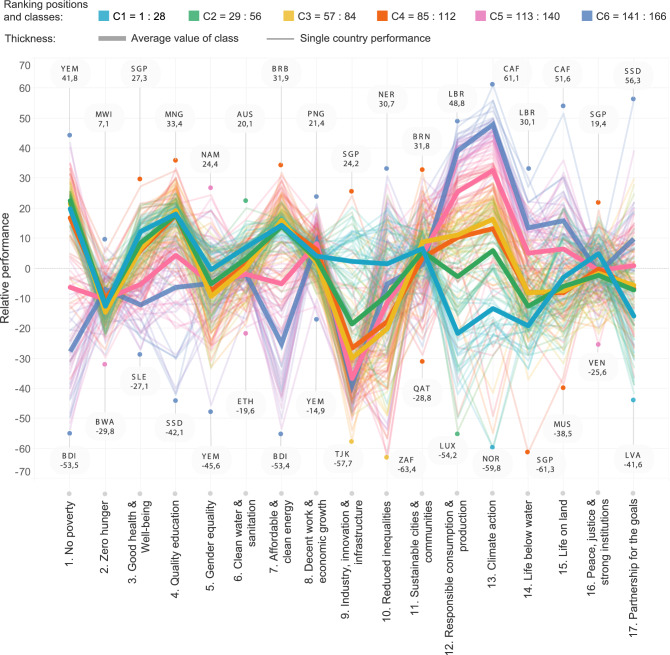
**The spectra of countries’ relative performances, obtained as** $P_{cg} - (k_c/17)$. Countries are first ranked and then clustered according to their average performance (i.e., the SDG Index or, equivalently, their degree). Based on the ranking positions, we define six classes of performance: light blue (countries in positions $1 - 28$), green ($29 - 56$), yellow ($57 - 84$), magenta ($85 - 112$), pink ($113 - 140$) and violet ($141 - 166$). The classes’ average spectra of relative performances are shown in thicker lines. The top and bottom relative performers in each Goal are pointed out, and their performance value is color-coded as their corresponding class. The figure has been generated using Tableau 2020.3, [https://www.tableau.com/].

The need for global centrality metrics to measure the complexity of the system clearly arises when considering the heterogeneity of countries’ performances across the Goals, as we address in Fig. 2. The figure plots countries’ performances as defined by the 2020 SDG Index and Dashboard^26^ (see “Materials and methods” section). In Fig. 2, countries are ordered according to their ranking position as defined by their degree (or, equivalently, the SDG Index). These countries’ performances (which from hereon we define as ‘spectra’) are relative ones, as they are obtained by subtracting the average performance of the countries, $k_c/17$ (i.e., their SDG Index), from the Goal-specific performance, $P_{cg}$. This allows one to compare relative Goals’ performances of all countries according to their efforts in sustainable development, thus identifying areas where countries are investing more/fewer efforts and disclosing differences in their strategies. At the same time, if compared with the spectra in absolute performance values $P_{cg}$ (see Figure S2), relative spectra help prevent the perception that countries with high average performance values (i.e., degree) have high-performance values across all development sectors. At a glance, the heterogeneity of the spectra stands out. Countries exhibit very contrasting behaviours among them and across the Goals, witnessing that the world is not moving as a unique ensemble toward the achievement of sustainable development. As mentioned, this is possibly due to the heterogeneity of countries contexts and challenges, as well as the differences in national strategies that possibly enhance such heterogeneity across SDGs. To group countries according to their degree $k_c$ can help understand these differences. In fact, Fig. 2 shows the existence of two limit behaviours of the 28 top and the 28 bottom performing countries according to the SDG Index (or degree), i.e., of classes 1 and 6, whose spectra are almost entirely out of phase. These dynamics are more evident within Goals of environmental performances and exploitation, from Goal 12 to 15. As the spectra clearly show, the first 28 best countries in degree (class 1 in light blue) are poorly engaging toward the achievement of SDG 12 and 13. In particular, Norway is the relative worst performer in Climate Action, a Goal in which the country performs almost $-60\%$ with respect to its SDG Index. Instead, there are many low-degree countries (class 6 in violet) whose relative performances in Climate Action are higher, with the Central African Republic (CAF) recording $+60\%$ of performance with respect to its SDG Index. Even if less accentuated, the spectra of top and bottom degree countries are also out of phase in SDG 17, the one invoking partnership. In this Goal, countries nearer to fulfil most of the Agenda are actually the worst relative performers (e.g., Latvia—LVA). Other examples of this out of phase behaviour of the countries in class 1 and 6 figure in correspondence of Goals 1, 2, 7 and 14 (Zero Poverty, Zero Hunger, Clean Energy and Life below Water, respectively). Drops of performances occur for top-degree countries in Goals 2 and 14, while for bottom-degree countries in Goals 1 and 7. For example, Singapore attainment of SDG 14 is $-60\%$ with respect to its average performance in sustainable development. Yemen stands as an exception of such a pattern since, in Goal 1, this country performs $40\%$ better than its average value (although this value may be a consequence of the assumptions on the data, see “Materials and methods” section).

The spectra depict the complexity of the variety of approaches toward sustainable development, in which the specificity of countries’ characteristics has its role in determining the attainment of the Goals. Therefore, we argue that analyses designed to consider and embed this complexity can shed new light on the state of the art in sustainable development. The introduction of network theory is a first step toward this direction, and it allows us to define novel aggregate scores based on a data-driven definition of the weights $w_g$ in Eq. (2). In particular, the introduction of network-comprehensive centrality measures may help explore different dimensions of the SDG topic (and consequently, countries’ status), and it allows one to define bottom-up weighting approaches naturally.

### A data-driven weighting of countries

A first revision of the degree centrality in bipartite networks consists in weighting the connection of the node proportionally to the centrality value of the node at the other edge. Therefore, countries connected to more central SDGs obtain a higher scoring value, and *vice-versa*. According to this rationale, to define the aggregate score $S_c$ in Eq. (2) entails setting $w_g = v_g$, where $v_g$ is the centrality value for Goal *g* and thus solving the system of coupled equations

3 $$\begin{aligned} {\left\{ \begin{array}{ll} S_c \propto \sum _g P_{cg} v_g, \\ v_g \propto \sum _c P_{cg} S_c. \end{array}\right. } \end{aligned}$$

Mathematically, the solution of this system is obtained by computing the so-called ‘singular vectors’ of the matrix $\mathbf {P}$ which determine the eigen-centrality vectors for countries and Goals, respectively^42^ (see “Materials and methods” section). While the degree is a local measure of centrality, the eigenvector is a global one, as it considers for the computation of the scores all possible links and strengths in the network^39–41^. However, as shown in Figure S1, the eigenvector centrality brings no further information in terms of rankings than the one by the degree centrality ($99.9\%$ in both Pearson’s and Spearman’s correlation measures, see Figure S1). This lack of added value is due to the intrinsic correlation that the degree and eigenvector centrality show when the spectral gap–i.e., the delta between the first and second largest singular value of the incidence matrix (see “Materials and methods” section)–is large^41^. For this particular bipartite network, the second largest singular value is roughly one fourth of the principal singular value, implying high correlation between the degree and eigenvector centrality^43^ (see “Materials and methods” section). Therefore, in the countries-SDGs network, the use of non-uniform weights as in Eq. (3) is almost ineffective in changing the point of view about the state of sustainable development, and other rationales about countries inter-plays with Goals must be introduced to remove the degree-bias that characterizes the eigenvector centrality^43^.

The use of the centrality metrics defined within the field of Economic Complexity (EC)^44–49^ can help in the characterization of more complex inter-plays between countries and Goals. Based on the data regarding the export baskets of countries, EC aims at determining the stage of innovation and competition countries find themselves at^44^. EC methods update the simplest proxy of innovation, i.e., the degree of countries in the bipartite system of trade, blamed for not considering the sophistication of the traded products^48^. In fact, the idea upon which EC theory is constructed is that, in a looping system, if a product is only exported by few countries, this item is more knowledge-intensive than other items exported by many other countries. (In EC, the word ‘knowledge’ intends knowledge of production, resources, human and capital investments, eventually^50^.) This fact determines higher EC scores of more knowledge-intensive goods.

In a similar manner, we can adapt the EC theory and methods to the network of countries and SDGs, therefore introducing new reasoning about how countries act in sustainable development. In tailoring the EC framework to the SDGs one, we assume that, if within a Goal only a few countries record near to optimal performance values, this Goal is more knowledge-intensive than the others, thus resulting in a higher EC score. Countries recording such optimal performances are those in more favourable conditions to attain the Goals. In fact, here, we translate ‘knowledge’ into policy and intervention designs and implementations; awareness and preparedness to face the challenges, all well-known factors for affecting countries performances in sustainable development^3,18,26,51–53^.

In this work, we adopt the *GENeralized Economic comPlexitY* framework, said GENEPY, which has been shown to reconcile the contrasting methodologies on economic complexity, and it is also a reliable method for processing non-binary incidence matrices as the one of the countries-SDGs bipartite system^48^. For the sake of clarity, in the following, the adaptation of the GENEPY framework to the context of the Agenda 2030 is defined as SDGs-GENEPY. To the best of our knowledge, Cho et al.^38^ is the only existing example in literature proposing to adapt EC methodologies and centrality metrics to score countries performances within the Agenda 2030. However, our work differs from that one in both methodology (the *Method of Reflection* from Hidalgo et al.^44^ is used, instead), and data, since that work is limited to the Asian Region.

The SDGs-GENEPY rationale defines two related centrality properties, $S_c$ for countries and $Y_g$ for SDGs, defined through the following system

4 $$\begin{aligned} {\left\{ \begin{array}{ll} S_c \propto \frac{1}{k_c} \sum _g P_{cg} \frac{Y_g}{k_g'}, \\ Y_g \propto \frac{1}{k_g'} \sum _c P_{cg} \frac{S_c}{k_c}, \end{array}\right. } \end{aligned}$$

in which $k_c = \sum _g P_{cg}$ is the degree of the countries, therefore the sum of all Goals’ performances (i.e., the value of the aggregate score supposing $w_g = 1$ for all SDGs). The term $k_g' = \sum _c P_{cg}/k_c$, that we define as ‘adjusted Goal’s degree’, is the degree of Goal *g* accounting for the relative performances of countries within it (relative performances of countries can either be computed as the subtraction of the average performances, as in Fig. 2, or using the ratio $P_{cg}/k_c$, and the same results and observations hold, see Figure S3). Therefore, to evaluate the aggregate score of countries’ statuses $S_c$ according to the SDGs-GENEPY requires computing the $Y_g$ values and it entails assuming, in Eq. (2), $w_g = Y_g/k_g'$ . Notice that, similarly to the eigenvector centrality, the metrics provided by the SDGs-GENEPY framework are also global ones since they account for the overall structure of the network^48^ (see “Materials and methods” section). Nevertheless, although the mathematical structure of Eq. (4) is formally an eigenvector one (see “Materials and methods” section), the resulting $S_c$ centrality metrics is no longer degree-dominated due to the division of the $S_c$ values by the degree $k_c$. A *toy model* to exemplify the difference of perspective offered by the SDGs-GENEPY approach is given in Section S1 in the Supporting Information.

A resume of the different weighting strategies for the Sustainable Development Goals that we adopted in this work is given in Table 1.

**Table 1:** Weighting approaches through different centrality metrics.

Centrality measure	Aggregate score	Weighting value
Degree	$S_c = \sum _g P_{cg}$	$w_g = 1$
Eigenvector	$S_c \propto \sum _g P_{cg} v_g$	$w_g = v_g$
SDGs-GENEPY	$S_c \propto \sum _g P_{cg} Y_g/k_g'$	$w_g = Y_g / k_g'$

In the formulas: $S_c$ is the aggregate score for country *c*, generally defined according to Eq. (2); $P_{cg}$ is the value of countries’ performances in Goal *g*; $w_g$ is the weighting value defined in Eq. (2); $v_g$ is the centrality score for SDGs according to the eigenvector centrality, Eq. (3); $Y_g$ is the centrality score for SDGs according to the SDGs-GENEPY framework, Eq. (4), and $k_g'=\sum _c P_{cg}/k_c$ is the adjusted Goals’ degree (see “Materials and methods” section).

**Figure 3:**
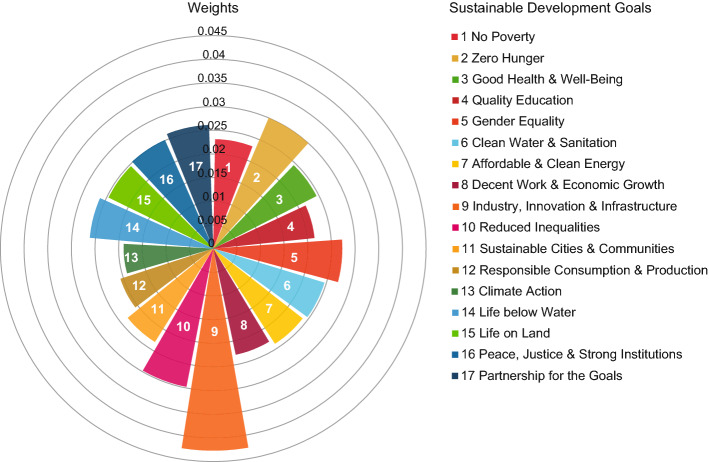
**The SDGs-GENEPY weights of the Sustainable Development Goals.** The radial bar chart plots the SDGs-GENEPY weights $Y_g/k_g'$ for all Goals (see “Results” section, Eqs. (4), and “Materials and methods” section). Conversely from the original SDGs circle (in which all Goals are equally distributed on a *doughnut plot*^11^), the present radial bar plot remarks that–although Goals are all equal in principle–there exists a difference of importance according to the proposed data-based approach. The figure has been generated using Excel 2016.

### A picture of global responses in sustainable development

Applying the economic complexity theory to the bipartite network of countries and SDGs provides useful insights about how countries are currently responding to the call for actions toward a more equitable, just, and sustainable future. We exemplify these results through the application of the SDGs-GENEPY framework on the data from the 2020 Dashboard by Sachs et al.^26^ (see “Materials and methods” section). Let us start from the results obtained from the computation of the SDGs-GENEPY values for Goals and, consequently, the weights $Y_g / k_g'$. In Fig. 3, the weighting values $Y_g / k_g'$ are shown. The top-weighted Goal is SDG 9 pertaining to innovation, followed by Zero Hunger and Reduced Inequalities, SDG 2 and 10, respectively. Climate Action (SDG 13) is the least weighted, preceded by SDGs 12 and 4, pertaining to sustainable consumption and education, respectively. The wide differences among the weights demonstrate that the SDGs-GENEPY framework is able to capture the contrasting performances among the top and bottom-ranked countries shown in Fig 2. In fact, this weighting of Goals reflects the poor performances by (generally) high performing countries in some SDGs (e.g., Norway in SDG 13, as will be further detailed; see, also Section S1 in SI). Moreover, these results provide one more piece of evidence that the SDGs are not equally integrated into national strategies worldwide. Consequently, the SDGs-GENEPY weighting values of less prioritised Goals are lower than that of more prioritised ones.

**Figure 4:**
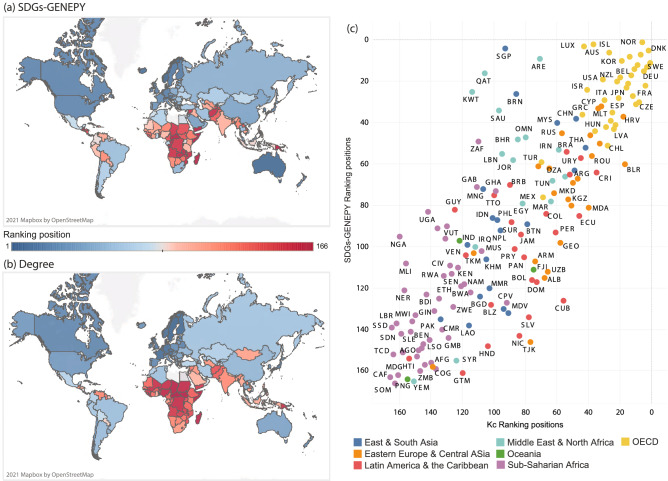
**Countries rankings according to the degree and SDGs-GENEPY values**. In panel (**a**), countries are coloured according to the ranking position computed by the SDGs-GENEPY score. Panel (**b**) shows the ranking position computed by the degree or, equivalently, the SDG Index^26^. The ranking position is defined in both maps according to descending score (1 = best performer, 166 = worst performer). In panel (**c**), we scatter the values of the two rankings: on the x-axis is the degree ranking, on the y-axis, the SDGs-GENEPY one. Countries are colour-coded according to their Region as specified in the legend, in accordance with the regional division in the 2020 Dashboard^26^. Countries lying along the diagonal share the same ranking position both in SDGs-GENEPY and SDG Index. The figure has been generated using Tableau 2020.3, [https://www.tableau.com/]; in panels (**a**) and (**b**), underlying maps are provided by OpenStreetMap, [https://www.openstreetmap.org].

Such a weighting approach determines the ranking of countries according to SDGs-GENEPY score, which differs from the one by the degree centrality (see Section S1). In Fig. 4, we map countries’ rankings according to the SDGs-GENEPY index and the degree value (panels (a) and (b), respectively); panel (c) resumes the differences between the two by scattering the ranking values, with countries colour-coded according to Regions, as defined in the 2020 Dashboard^26^ (see “Materials and methods” section). As the Figure shows, although the two rankings are mostly aligned (Pearson’s correlation coefficient 0.81), significant differences arise. As most remarkable examples, we cite here: Singapore (SGP), which jumps from the lower half of the chart to the top of it, moving from position 93 in degree to position 4 in the SDGs-GENEPY $S_c$; and South Africa (ZAF), moving from 110 in degree to 49 in the SDGs-GENEPY score. Other examples include Chile (CHL), moving from the 28-th position in degree to the 51-th in the SDGs-GENEPY score, and Cuba (CUB), which downgrades from the 56-th place in degree to the 126-th in SDGs-GENEPY $S_c$.

To explain the reasons behind these variations, we refer to Norway as a relevant example: Norway is among the absolute top performers within SDG 9 (having the largest weighting value $Y_g/k_g'$), together with South Korea and Singapore. Most countries perform poorly within this Goal–only $50\%$ of the countries is above the $40\%$ of Goal achievement –, as also represented in Fig. 2. Consequently, the SDGs-GENEPY framework assigns a higher weight to countries that are better performers in this Goal. Also, Norway figures as the best absolute performer in Goal 10 and reaches good performances in Goal 2, thus explaining the upgrading of the North-European country from the sixth to the first position in the SDGs-GENEPY $S_c$ ranking. Another relevant example is represented by the case of Singapore, a nation that, due to its outstanding performances in more knowledge-intensive SDGs, has reached the third position in SDGs-GENEPY. In contrast, Norway and Singapore are among the worst relative performers in SDGs 13 and 12, respectively (see Fig. 2). Still, their low performances in these SDGs are comparatively less relevant within the SDGs-GENEPY framework due to the lower weight values assigned to these two Goals.

## Discussion

Defining aggregate scores in sustainable development is a recurrent problem, which needs to be addressed to track the path toward achieving the Goals within the Agenda 2030. Many strategies can be pursued for their computation (see, e.g.,^25,26^); nevertheless, the complexity of the Agenda 2030 should not be neglected when defining aggregate scores. In light of this complexity, in this work, we have introduced a novel perspective on sustainable development in which we addressed–within a network science framework–the need for ranking countries for their status concerning the Agenda. In particular, we show that the countries-SDGs system can be structured as a bipartite network and that, by using the centrality tools, different weighting approaches naturally emerge for the computation of aggregate scores to rank countries.

Thanks to this network representation of the system, we show that the SDG Index identified by Sachs et al.^26^–which, in line with the Agenda’s principles, considers equal weights for all Goals corresponds to measure the degree of countries. In network science, the degree centrality measures the local behaviour of the node, and it does not account for the complex interconnections of the system^41^. A first step toward the use of global metrics to account for the network structure is the use of the eigenvector centrality. However, we have demonstrated that the degree and eigenvector centrality in this countries-SDGs system substantially carry the same information. Besides the formal reasoning about the spectral gap, the strong correlation between the two centrality metrics is due to the fact that countries’ performances in SDGs are mutually correlated (see Figure S4). This fact highlights that countries set in similar development conditions (*sensu*, Baldwin et al.^54^) tend to emulate each other performances^55^, and it explains why, when ranked for their degree, nearby ranking-positioned countries show similar behavioural patterns (see Fig. 2 and Figure S4). Nevertheless, heterogeneity of countries’ performances beyond their average value (or equivalently, the degree) is clear from Fig. 2. This phenomenon suggests the need for more subtle metrics able to unravel the complexity of the system, a need we address through the *GENeralized Economic comPlexitY* framework (SDGs-GENEPY)^48^.

The SDGs-GENEPY approach we propose for the creation of one aggregate score brings two main positive advancements. Firstly, the weights $w_g = Y_g / k_g'$ are self-emerging from the data, and they account for the relative performances of countries as measured by term $k_g'$, thus providing a data-driven embedding of the synergies and trade-offs across the SDGs. Secondly, the division of the SDGs-GENEPY $S_c$ values for the degree of countries $k_c$–intrinsic of the computation of the index–removes the undesired degree-bias which is known to affect eigenvector-based centrality measures^43^, thus providing useful insights about the countries’ status in sustainable development. These characteristics of the SDGs-GENEPY framework can be interpreted in light of some further considerations concerning the Agenda 2030. The adjusted degree values $k_g'$ are determined by the relative performance values $P_{cg}/k_c$. In particular, the term $k_g'$ is larger if, in a given Goal *g*, there are many large performance values $P_{cg}$ recorded in countries at low degree $k_c$ values. Considering that the weights $w_g$ are inversely proportional to the adjusted degree, it follows that heavier (in the sense of weights) Goals are also those that top-performing countries favour to the detriment of other Goals (see SI, Section S1). The fact of having found Climate Action and Innovation as, respectively, the lowest and greatest weighted Goals, witnesses such result, since the $w_g = Y_g / k_g'$ values are mainly determined by the relative performances of high-income and sustainable-outperforming countries, such as Norway (see Fig. 2).

In fact, evidence of the validity of such analysis can be found in Norway’s development strategies, among the most relevant examples in this study. Norway is currently diversifying its industrial sector by enhancing investments in the Research and Development area, so to face the reduction in prices of crude oil^56,57^ (see, also, the Climate Action Tracker, [https://climateactiontracker.org/countries/norway/]). Norway is one of the worldwide leader exporters of crude oil^58^, a fact that puts under the spotlight the country’s shared responsibility in Climate Action and the permanent presence of trade-offs between economic and environmental issues at the world level^59^. Therefore, in the SDGs-GENEPY indexing approach, the heterogeneity of countries and contrasting policy implementations are naturally embedded through the data and brought up by the algorithm, determining the weights of SDGs. This hierarchy testifies the shared global responsibility in sustainable development and the intrinsic compromise among political willingness, opportunities and capacities to move toward sustainable development^26,60,61^. This compromise is even more evident in countries with more favourable conditions to fulfil the Agenda, resulting in higher ‘knowledge’ (i.e., policy and intervention designs and implementations; awareness and preparedness to face the challenges^3,18,26,51–53^).

In light of these considerations, we can interpret the SDGs-GENEPY ranking of countries as a picture of shared responsibilities, where it emerges the possibility for nations to act as role-models and promote the achievement of global sustainable development. In light of the emulation phenomena among countries^55^, we argue that identifying role-model countries is rather relevant, and it can pave the way to a new strategy for boosting sustainable development in the next decade. In particular, our ranking can be used as an ‘*ex post*’ and complementary tool to the Rapid Integrated Assessment–RIA–analysis^2^ which the United Nations conduct to monitor the willingness of countries in integrating the Goals within their national strategies. In this sense, our analysis would effectively provide insights about the implementation of such plans, also providing a tool for comparing the efforts across countries. Moreover, such an approach can be suitably adapted to the sub-national level by using regional data on sustainability performance, thus revealing crucial features of countries’ regional efficiency in sustainable development.

In conclusion, we acknowledge the complexity of the system defined within the Agenda 2030. In light of such complexity, and for the sake of providing more complete analysis, we address future work to embed models of interaction across the sustainable development sectors (e.g., those related to the complex interaction between water, energy, and food^62^ among the many^12,15,16,22,63^). Moreover, the burgeoning literature in the field of SDGs assessments suggests the presence of different ideologies about how to properly measure the status of countries along their sustainable development path. We realise that the understanding of such paths should not be shrunk to a single indicator analysis. Therefore, to fully understand countries’ paths toward sustainable development, we suggest using different and complementary mathematical approaches, such as e.g., the computation of both the degree and SDGs-GENEPY rankings. The adoption of more classical methods (such as the degree-like ones) combined with the SDGs-GENEPY would provide a bird’s-eye view of the conditions of countries to achieve sustainable development while providing a list of change-making places and actions that can help meet the 2030 deadline.

## Materials and methods

### Data

Notwithstanding the call for efforts toward the standardisation in the data collection by all National Statistical Systems, NSSs, launched by the Cape Town Global Action Plan in 2017^64^, the data accessible at the UN Statistics Division (available at https://unstats.un.org/sdgs/indicators/database/) clearly show that work is still needed to have a comprehensive, homogeneous, and extensive database covering all countries and years under the Agenda 2030 and beyond. For this reason, the input data we are using are taken from the 2020 SDG Index and Dashboard^26^, which represent a commendable step forward in data collection, homogenisation and assessment of countries progress in sustainable development. The aim of the Dashboard is to provide yearly rankings of UN countries based on an aggregate score of all Goals’ performances. The score is intended to be readable as a percentage of achievement of all the Goals, ranging from 0 to 100; therefore, countries close to 100 are approaching the complete fulfilling of the Agenda’s Goals according to the indicators used to compute the score^36^. The score is constructed upon a number of indicators providing quantitative information about countries performances. All listed indicators are normalised according to an optimum and a minimum value of indicator performance to ensure comparability and aggregation of measurements (we refer the reader to^26,36^ for further details). Listed indicators are updated every year, accounting for advances in monitoring and research. In order to provide statistical-sound results, we only refer to 2020 data, thus not inferring any possible missing data back in other years’ Dashboards. The 2020 dataset constitutes of 115 indicators across the Goals, 30 of which are specifically defined for the members of the Organization for Economic Co-operation and Development (OECD). The Dashboard only includes countries covering at least $85\%$ of the indicators, totalling 166 out of 193 UN countries. To have OECD-specific indicators entails that, with respect to the same Goal *g*, the term $N_{cg}$ (from which, in Eq. (1), the value of performance $P_{cg}$ is obtained) differs between OECD and other countries. The Dashboard also introduces Regional scores, assigning countries to 7 different Regions around the world, namely: Sub-Saharan Africa, Middle East and North Africa–MENA–, East and South Asia, Eastern Europe and Central Asia, Latin America and the Caribbean–LAC–, Oceania and OECD group, which we use to colour-code countries in Fig. 4.

In line with the methodology exemplified with the SDG Index, we replace countries’ missing data with the Regional score in that same Goal^36^. Notice that, as pointed out by the Authors of the Dashboard^65^, this assumption might have implications in any analysis.

### Eigenvector centrality

Let $u_c$ be the eigencentrality of country *c* and $v_g$ the eigencentrality of Goal *g*. By definition, the eigencentrality value for country *c* is its degree weighted by the centrality of all Goals, and *vice-versa*^39^. In this work, the centrality score for countries $u_c$ coincides with the computation of $S_c$ when setting $w_g = v_g$ in Eq. (2). The computation of the eigenvectors of a matrix requires the matrix to be squared. Incidence matrices of bipartite networks, such as the matrix $\mathbf {P}$ in this work, are rectangular, instead. In order to compute the eigenvector centrality of countries and Goals, the matrices $\mathbf {A}=\mathbf {PP'}$ and $\mathbf {B}=\mathbf {P'P}$ are introduced, where $\mathbf {P'}$ is the transpose of the matrix $\mathbf {P}$^42,66^. The system in Eqs. (3) can hence be solved in closed form as

5 $$\begin{aligned} {\left\{ \begin{array}{ll} \sigma _1^{2} \mathbf {u}_1 = \mathbf {A u}_1, \\ \sigma _1^{2} \mathbf {v}_1 = \mathbf {B v}_1, \end{array}\right. } \end{aligned}$$

in which the term $\sigma _1$ is the largest singular value of the matrix $\mathbf {P}$^66^, or, equivalently, the square root of the largest eigenvalue $\lambda _1$ of the matrices $\mathbf {A}$ and $\mathbf {B}$. The vector $\mathbf {u}_1$ and $\mathbf {v}_1$ are the singular vectors of the matrix $\mathbf {P}$ associated to $\sigma _1$ or, equivalently, the eigenvectors of $\mathbf {A}$ and $\mathbf {B}$ associated to the largest eigenvalue $\lambda _1$^42,66^. Notice that, due to the mutual relationship between eigen- and singular values, the spectral gap can be equivalently measured between the two largest eigenvalues of the matrices $\mathbf {A}$ and $\mathbf {B}$ or between the singular values of the matrix $\mathbf {P}$.

### The SDGs-GENEPY framework

The *SDGs-GENeralized Economic comPlexitY* scoring and weighting approach is set in a linear algebra framework. The SDGs-GENEPY framework aims to define two properties $X_c$ for countries and $Y_g$ for SDGs, which can account for the EC rationale and so embed the inter-play between countries and Goals. In this rationale, a Goal is more knowledge-intensive if only a few countries–also in more favourable conditions to attain the Goals, see “Results” section and Section S1–record near to optimal performance values. These countries are those with a higher change-making power and more responsible for prioritising certain Goals. This fact can be mathematically obtained by defining the system in Eqs. (4). Similarly to the eigenvector centrality, a closed solution for this system is provided by solving the coupled singular vectors $\mathbf {X}$ and $\mathbf {Y}$ associated to the largest singular value $\sigma _1$ of the matrix $\mathbf {W}$ defined as

$$\begin{aligned} W_{cg} = \frac{P_{cg}}{k_c k_g'}. \end{aligned}$$

The matrix $\mathbf {W}$ helps in defining the EC rationale and in providing a symmetric representation of the bipartite system for which the $\mathbf {X}$ and $\mathbf {Y}$ are determined. In fact, the vector $\mathbf {X}$ for countries is the eigenvector of the largest eigenvalue of the matrix $\mathbf {N}$ defined as

6 $$\begin{aligned} N_{cc^{*}} = \mathbf {WW'} = \sum _g \frac{P_{cg} P_{c^{*}g}}{k_c k_{c^{*}} (k'_g)^2}; \end{aligned}$$

the vector $\mathbf {Y}$ for SDGs is the eigenvector of the largest eigenvalue of the matrix $\mathbf {Z}$ defined as

7 $$\begin{aligned} Z_{gg^{*}} = \mathbf {W'W} = \sum _c \frac{P_{cg} P_{cg^{*}}}{k_c^2 k'_g k'_{g^*}}. \end{aligned}$$

In this work, the centrality score for countries $X_c$ coincides with the computation of SDGs-GENEPY $S_c$ values, when setting $w_g = Y_g/k_g'$ in Eq. (2).

For further details, we refer the readers to Sciarra et al.^48^ for a complete description of the algebra beyond the framework. However, some comments are due to the readers to follow along the reasoning behind this work entirely. Thanks to the differences in the input bipartite system, adapting the GENEPY framework to the Agenda 2030 (i.e., the SDGs-GENEPY we introduced in this work) provides a more straightforward mathematical rationale than the one presented in the original work for trade. Building upon the export data, the GENEPY index in^48^ is a multidimensional centrality score for economic complexity in which two eigenvectors of the matrix $\mathbf {N}$ for countries are combined in quadratic form (or, $\mathbf {Z}$ for SDGs). Without any loss of information, in this work, we limit our analysis to the first eigenvectors of the matrices $\mathbf {N}$ and $\mathbf {Z}$, for countries and Goals, respectively. In fact, the eigenvectors associated with smaller eigenvalues bring no relevant added information, and their quadratic terms in the formulation of the SDGs-GENEPY score can be neglected (see Figure S5). Moreover, the diagonal values of the matrices $\mathbf {N}$ and $\mathbf {Z}$ are left as computed following Eq. (6) and Eq. (7), respectively (differently from the export case, the diagonal values do not bias the results, see Figure S6). Finally, with respect to the trade case, a further difference is that the incidence matrix of the bipartite network of countries and SDGs defines non-binary, so weighted, connections among the nodes.

## Supplementary Information

Supplementary Information.

## Data Availability

The data on countries performances in sustainable development supporting the findings of this study are freely available at [https://www.sdgindex.org/]. Other results are available from the authors upon request.

## Code availability

The code for the computation of the GENEPY index is publicly and freely available at [https://zenodo.org/record/3876721].
