# Supplementary material for: A network approach to rank countries chasing sustainable development
Source: Sci Rep. 2021 Jul 29;11:15441. doi: 10.1038/s41598-021-94858-2 (PMC8322206; doi:10.1038/s41598-021-94858-2)
Supplement: Supplementary file 1 — Supplementary Information. [file 41598_2021_94858_MOESM1_ESM.pdf]

Supporting Information for

A Network Approach to Rank Countries Chasing Sustainable Development

Carla Sciarra[1]*, Guido Chiarotti[1], Luca Ridolfi [1] and Francesco Laio[1]

[1]Department of Environmental, Land and Infrastructure Engineering, Politecnico di Torino, Corso Duca degli Abruzzi 24, 10129 Torino, Italy

*corresponding author: carla.sciarra@polito.it

Introduction

In this Supporting Information we provide supporting descriptions and figures about the mathematical results described in the main text. All supporting material has cross-reference in the main text.

References

[1] Sachs, J. D., Schmidt-Traub, G., Kroll, C., Lafortune, G., Fuller, G., & Woelm, F. (2020). Sustainable Development Report 2020.

[2] Sciarra, C., Chiarotti, G., Ridolfi, L., & Laio, F. (2020). Reconciling contrasting views on economic complexity. *Nature Communications*, 11(1), 1-10.

Section S1: A Toy Model to Detail the SDGs-GENEPY Approach

Let $\mathbf{A}$ be a $5\ x\ 4$ matrix with values A_{cg} ranging within the values $[0, 100]$ and defining the performance of a country c in each Goal g (see Table S1). Countries show very heterogeneous performances: most countries have difficulties in achieving Goal 3, except for country D. This latter country is almost the best performer across the Goals, and it loses ground in the achievement of Goal 4, in which all other countries are better performers. Instead, country B, which has very poor performance values across all Goals, has the primacy in achieving Goal 4.
The first analysis of this system is performed by measuring the degree of countries and Goals, i.e., by computing the values $k_c = \sum_g A_{cg}$, for countries, and $k_g = \sum_c A_{cg}$. The highest degrees are found for country C and Goal 4, respectively (see Table S1). Clearly, these metrics blind-sight the contrasting behaviours of countries, and the top-ranked Goal is the same in which the almost top-performing country performs worst (country D). Notice that the degree entails considering that all countries and Goals contribute equally (implicit weight $w_c = w_g = 1$) in the definition of the score for Goals and countries, respectively.

The SDGs-GENEPY approach proposed in this work can help to account for the heterogeneity of countries, and it provides a data-driven definition of the weights assigned to each Goal. Weights are defined mathematically by how countries are performing within the Goals. The SDGs-GENEPY framework comes from the economic complexity, and it represents a rationale obtained to reconcile the contrasting methodologies of economic complexity [5]. The mathematical framework introduces three terms (see Material and Methods section in the main text):

- the adjusted Goals' degree, i.e., the values $k'_g = \sum_c \frac{A_{cg}}{k_g}$.
- the SDGs-GENEPY score of countries S_c and Goals, Y_g, respectively.
- the weights of Goals in the computation of the score S_c, defined as $w_g = \frac{Y_g}{k'_g}$.

Although these terms are defined outside the analysis presented in this work, we can interpret them in this context of analysis. In particular:

- the adjusted Goals' degree, i.e., $k'_g = \sum_c \frac{A_{cg}}{k_c}$, measures the degree of the Goals accounting for the relative performances of countries within them. Due to the division of the A_{cg} values by the k_c ones, and differently from the standard degree k_g, the adjusted degree embeds global information about the system, in which each country's contribution to the degree is normalized with respect to the overall outcome of the country. In particular, the term k'_g is larger if there are many large performance values A_{cg} recorded in countries at low k_c values.
- the SDGs-GENEPY score of countries S_c and Goals, Y_g, are the final centrality scores. The scores are computed through a system of equations. The Y_g values are functional to the computation of the S_c ones, and vice-versa. In particular, the Y_g define the weights $w_g = Y_g/k'_g$ of the Goals in the computation of the score S_c. The weights are also inversely proportional

to the k'_g values, therefore, the higher the adjusted degree, the lower the weight of the Goal. Hence, we can interpret the term $1/k'_g$ as a measure of the global limitations in achieving the Goal.

Performing the SDGs-GENEPY framework onto the example matrix $\mathbf{A}$, the added value of our approach arises by looking at the values of the scores and weights, and their corresponding rankings (Table 1). To use the S_c SDGs-GENEPY values for ranking countries changes the perspective: Country D moves from position 2 in degree, to position 1 in SDGs-GENEPY. As stated in the main text, the framework captures the fact that country D is the only one able to achieve most of the Goals, except for Goal 4. This fact unavoidably downgrades the weight of Goal 4, which is only achieved by averagely worse-performing countries. Instead, the top-weighted Goal is Goal 3, in which only two countries, country C and D, record performance values higher than 50%. The ranking obtained through the Y_g values, might be interpreted as a ranking of Goals from the most to the least knowledge-intensive Goals; we interpret the concept of knowledge as policy interventions and designs, as well as awareness, willingness, and preparedness to face the challenges. Therefore, in this Toy Model, Goal 3 results being more knowledge-intensive than the others. Considering these results, the ranking obtained through the S_c values can be interpreted as a ranking of responsibility to drive sustainable development at the world level, fostering change-making actions in countries closer to the fulfilment of the Agenda.

Further comments

As it emerges from Toy Model example, as well as from Eqs (3) – (4), our work introduces a change of perspective for the weighting of the Goals. Compared to the most classical approach, i.e., considering all Goals to be equally weighted by countries, the SDGs-GENEPY framework provides a data-driven definition of these weights. Here, the weights arise as the combination of the values Y_g and k'_g. Through a complex network approach, the Y_g values define the centrality of the Goals accounting for the performance score S_c of all countries, and vice-versa. Looking at the system of equations (4), this double projection (from Y_g to S_c and vice-versa) occurs through the introduction of a functional kernel identified by the values $1/k'_g$, for the S_c values, and $1/k_c$ for the Y_g ones. Therefore, although necessary, the Y_g values are not sufficient to unravel alone the importance of the Goals within a given country, and the division for the k'_g values is required. Moreover, to rank the Goals according to the Y_g/k'_g values, rather than the Y_g values alone, can lead to different results. In the Toy Model, the information about the ranking of the Goals the two metrics bring is the same, but this is just a case. By comparing the rankings obtained from Y_g values with the one from the w_g values, Figure S7 in this Supporting Information, clear differences emerge (see, e.g., Goals 3 and 4). These differences are the results of the division for the k'_g values, which adds non-trivial information about the complex behaviour of countries in the network.

Table S1: Toy Model of the countries-SDGs system and outcomes of the analyses from the degree and SDGs-GENEPY framework. Countries' results are given in the top-right part of the table, while Goals' ones in the bottom-left part of the table. The numbers in brackets define the ranking positions of the values, from top to bottom-ranked (positions 1 to 5 for countries, 1 to 4 for Goals, respectively).

Country \ Goal	1	2	3	4	Countries: k_c degree (ranking)	Countries: S_c SDGs-GENEPY score (ranking)
**A**	43.91	57.92	8.38	96.75	206.97 (4)	0.37 (4)
**B**	0	48.54	3.57	99.67	151.78 (5)	0.31 (5)
**C**	96.67	65.83	69.50	74.74	306.76 (1)	0.50 (2)
**D**	99.48	82.81	91.28	20.93	294.51 (2)	0.57 (1)
**E**	56.51	75.59	22.89	76.20	231.20 (3)	0.42 (3)
**Goals**						
**k_g degree (ranking)**	296.58 (3)	330.70 (2)	195.64 (4)	368.30 (1)		
**k'_g adjusted degree (ranking)**	1.10 (3)	1.42 (2)	0.69 (4)	1.76 (1)		
**$w_g = Y_g/k'_g$ SDGs-GENEPY weights (ranking)**	0.47 (2)	0.33 (3)	0.80 (1)	0.23 (4)		
**Y_g SDGs-GENEPY score (ranking)**	0.53 (2)	0.47 (3)	0.56 (1)	0.42 (4)		

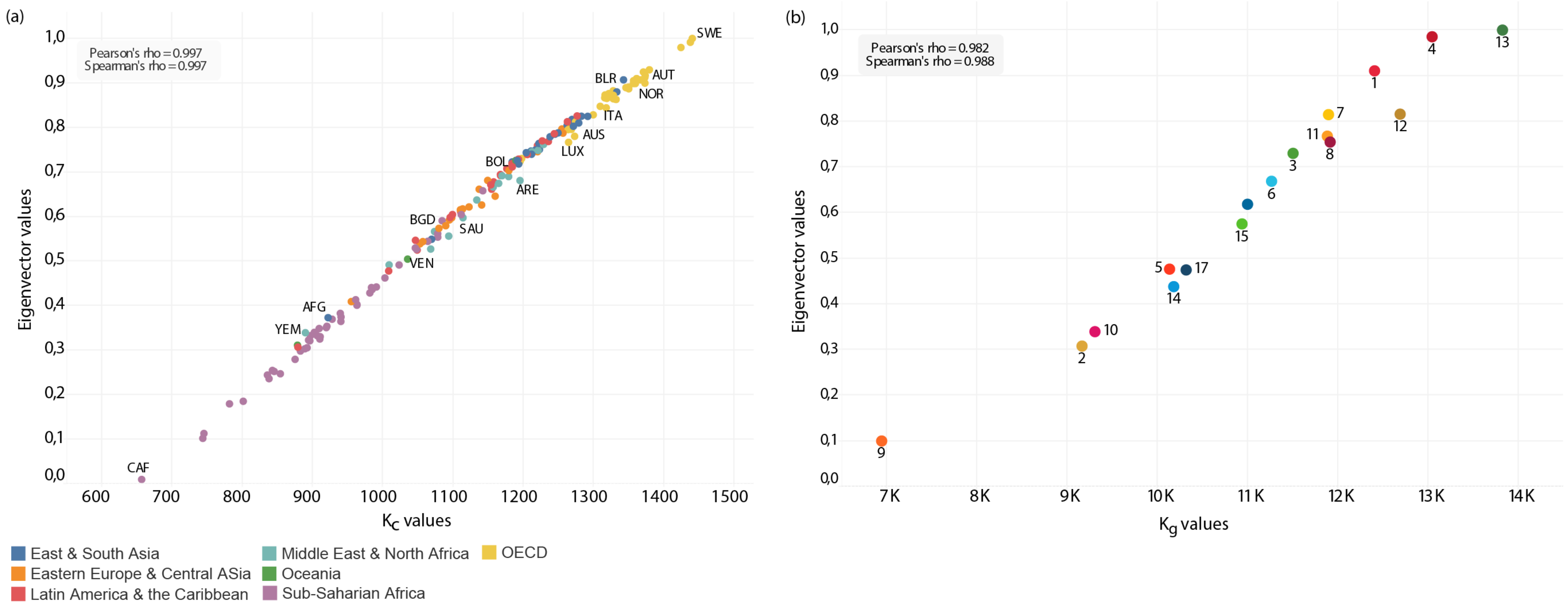

Figure S1: Scatter plot of the centrality values obtained by the degree and eigenvector measures. Panel (a) and (b) scatter the degree and eigenvector values of countries and Goals, respectively. For countries and Goals, the degree is defined as $\boldsymbol{k_c} = \sum_g \boldsymbol{P_{cg}}$ and $\boldsymbol{k_g} = \sum_c \boldsymbol{P_{cg}}$, respectively. The eigenvector score is obtained by computing the vectors $\mathbf{u}$ and $\mathbf{v}$, for countries and Goals, respectively, according to Eqs (5) – see Materials and Methods in the main text. In panel (a), countries are colour-coded according to their Region, as defined in [1]. In both panels, the eigenvector values are normalized between zero and one; the Pearson's and Spearman's correlation coefficients are specified (notice that the Spearman's one is a ranking-based correlation; thus it also provides information about the correlation between the two rankings).

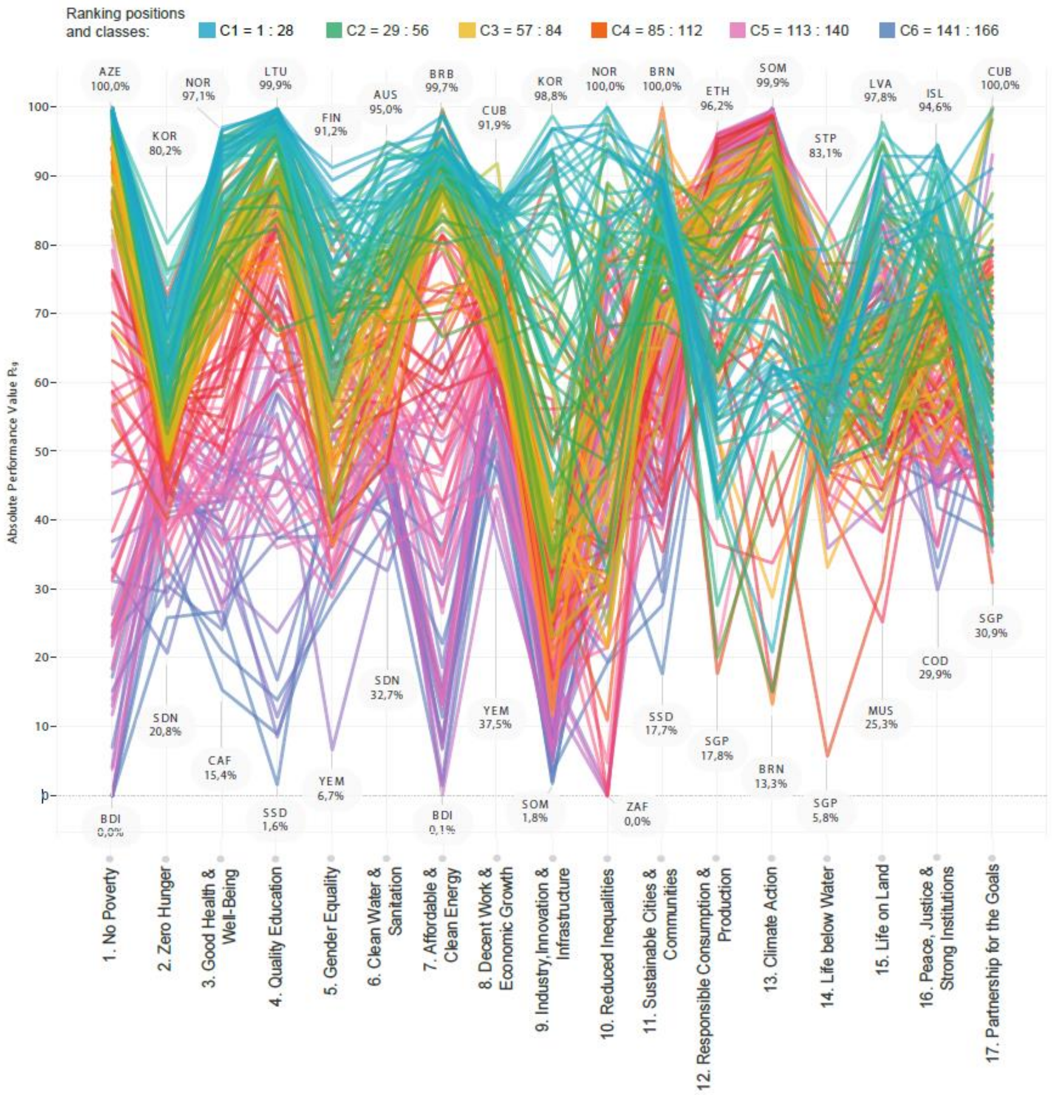

Figure S2: Spectra of the absolute performances of countries P_{cg}. Countries are first ranked and then clustered according to their average performance (i.e., the SDG index or, equivalently, their degree). Based on the ranking positions, we define six classes of performance: light blue (countries in positions 1 – 28), green (29 – 56), yellow (57 – 84), magenta (85 – 112), pink (113 – 140) and violet (141 – 166). The top and bottom absolute performers in each Goal are pointed out.

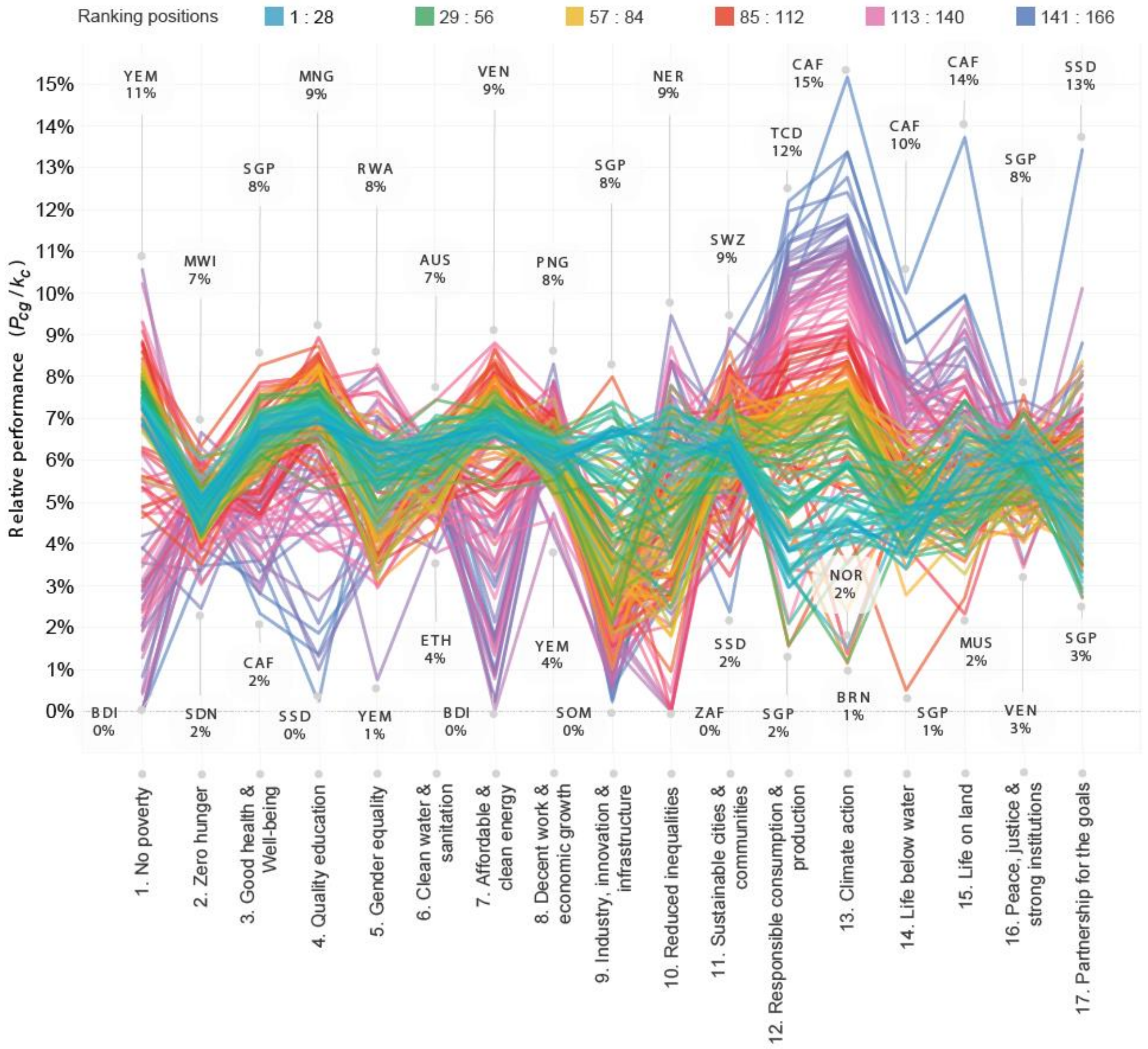

Figure S3: Spectra of the relative performances of countries obtained from the ratio P_{cg}/k_c. Countries are first ranked and then clustered according to their average performance (i.e., the SDG index or, equivalently, their degree). Based on the ranking positions, we define six classes of performance: light blue (countries in positions 1 – 28), green (29 – 56), yellow (57 – 84), magenta (85 – 112), pink (113 – 140) and violet (141 – 166). The top and bottom relative performers in each Goal are pointed out.

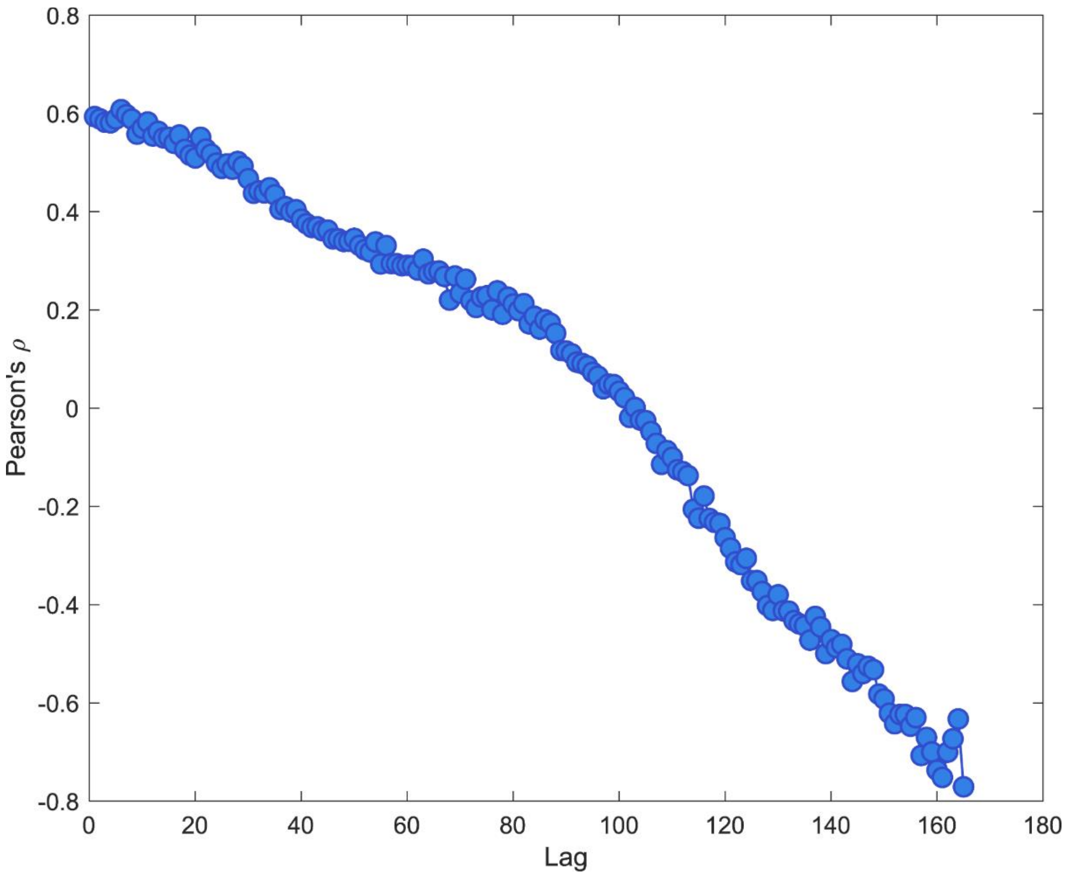

Figure S4: Pearson's correlation values of countries' performances. We consider the correlation between the rows of the matrix of performances $\mathbf{P}$. The rows of the matrix, and so countries, are ordered – top to bottom – according to decreasing value of countries' degree, $\boldsymbol{k_c}$. This fact allows one to order countries according to the similarities in their development conditions. For the sake of representation, we summarize correlation through the computation of lagged correlations: each point on the plot represents the average value of all correlations between countries positioned at a defined lag step. Therefore, the last point of the plot defines the correlation between the first and last rows (i.e., countries), for which a negative correlation value is obtained.

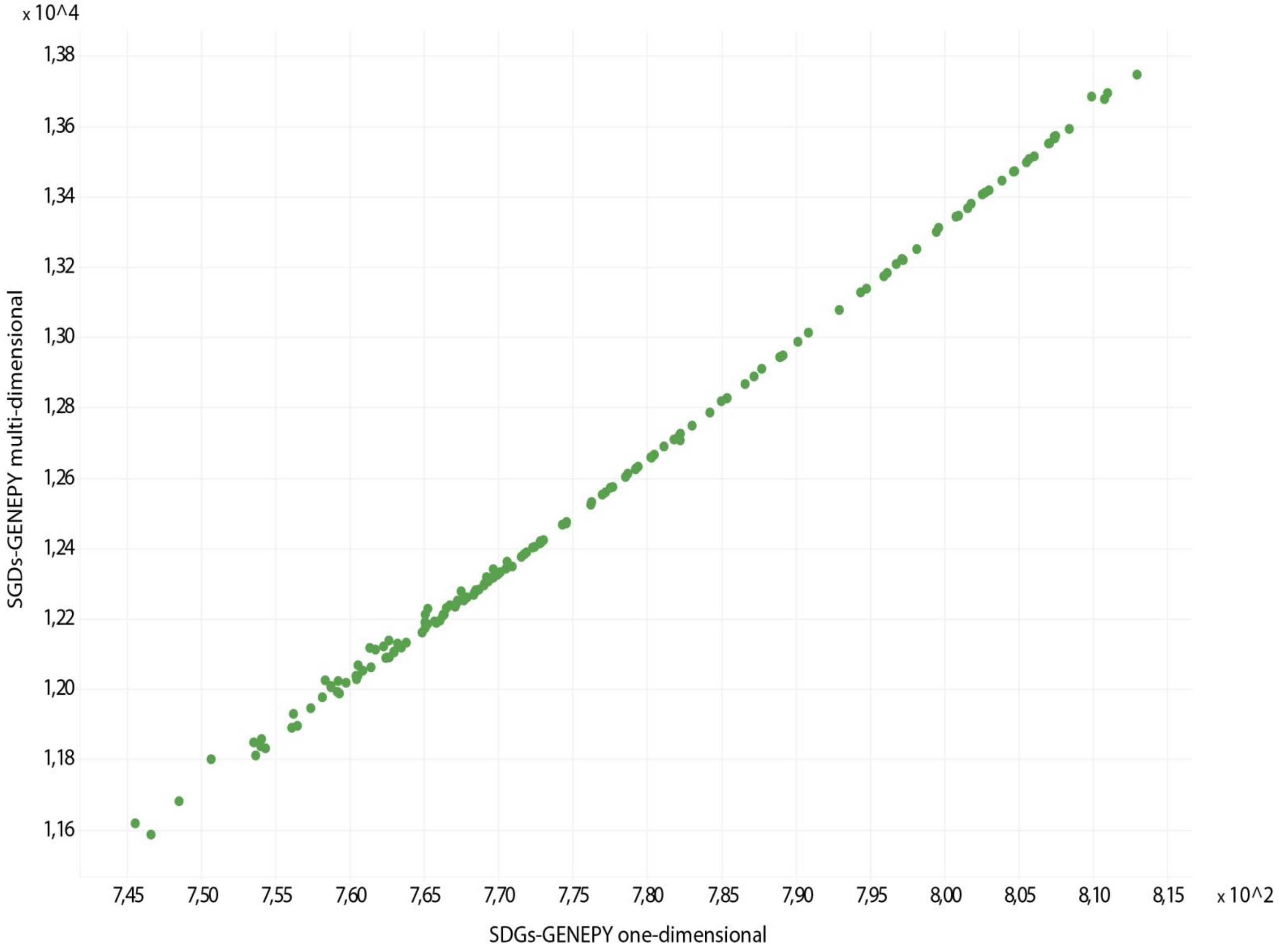

Figure S5: Scatter plot between the countries' centrality values computed through the SDGs-GENEPY one-dimensional score, Eqs. (4) in the main article, and the SDGs-GENEPY multi-dimensional index as mathematically defined in Sciarra et al., 2020, [2] and evaluated for the bipartite countries -- SDGs system.

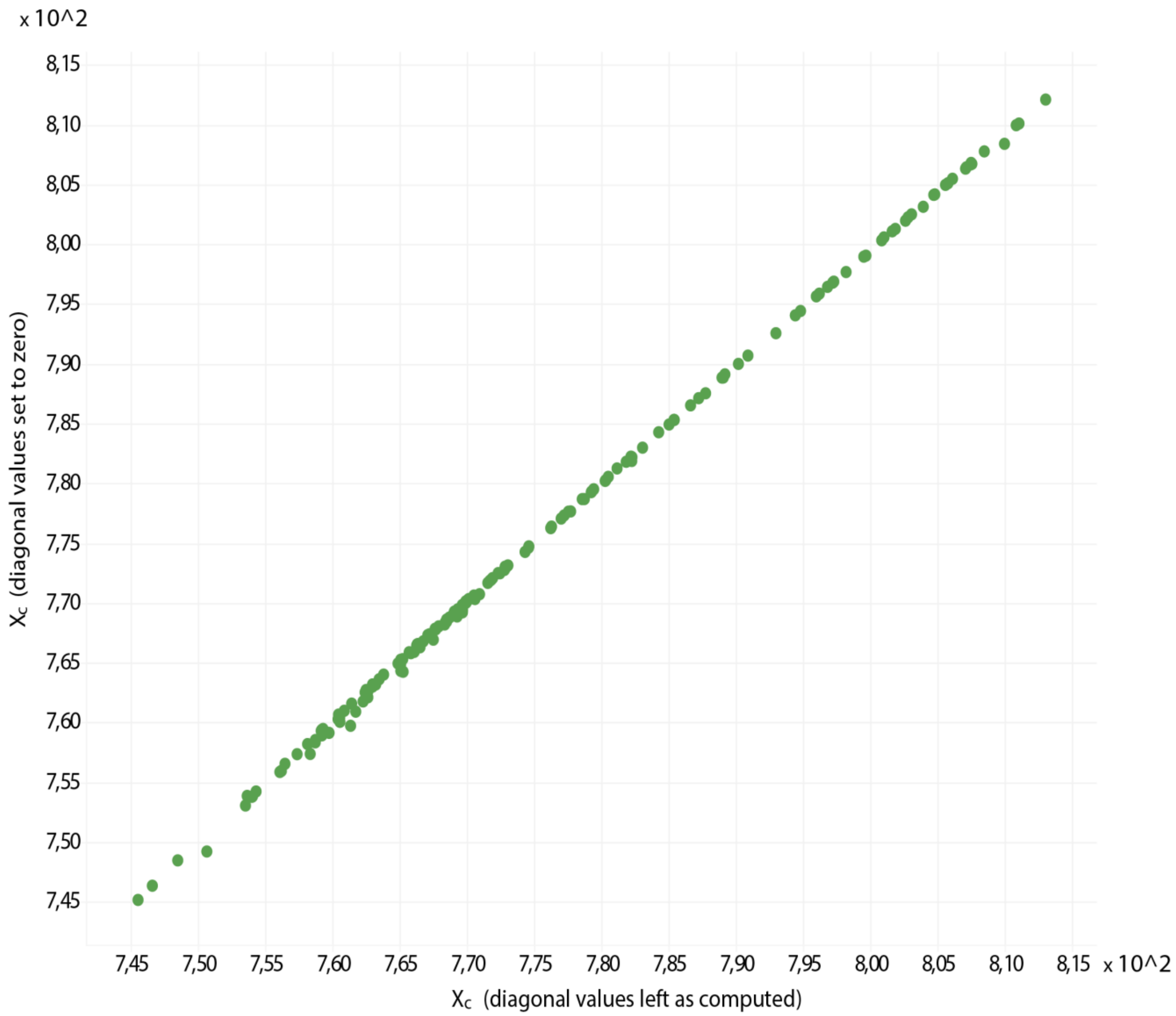

Figure S6: Scatter plot between the values X_c of the SDGs-GENEPY framework, Eqs. (4), computed either or not setting the diagonal values of the matrix $\mathbf{N}$, Eq. (6), to zero.

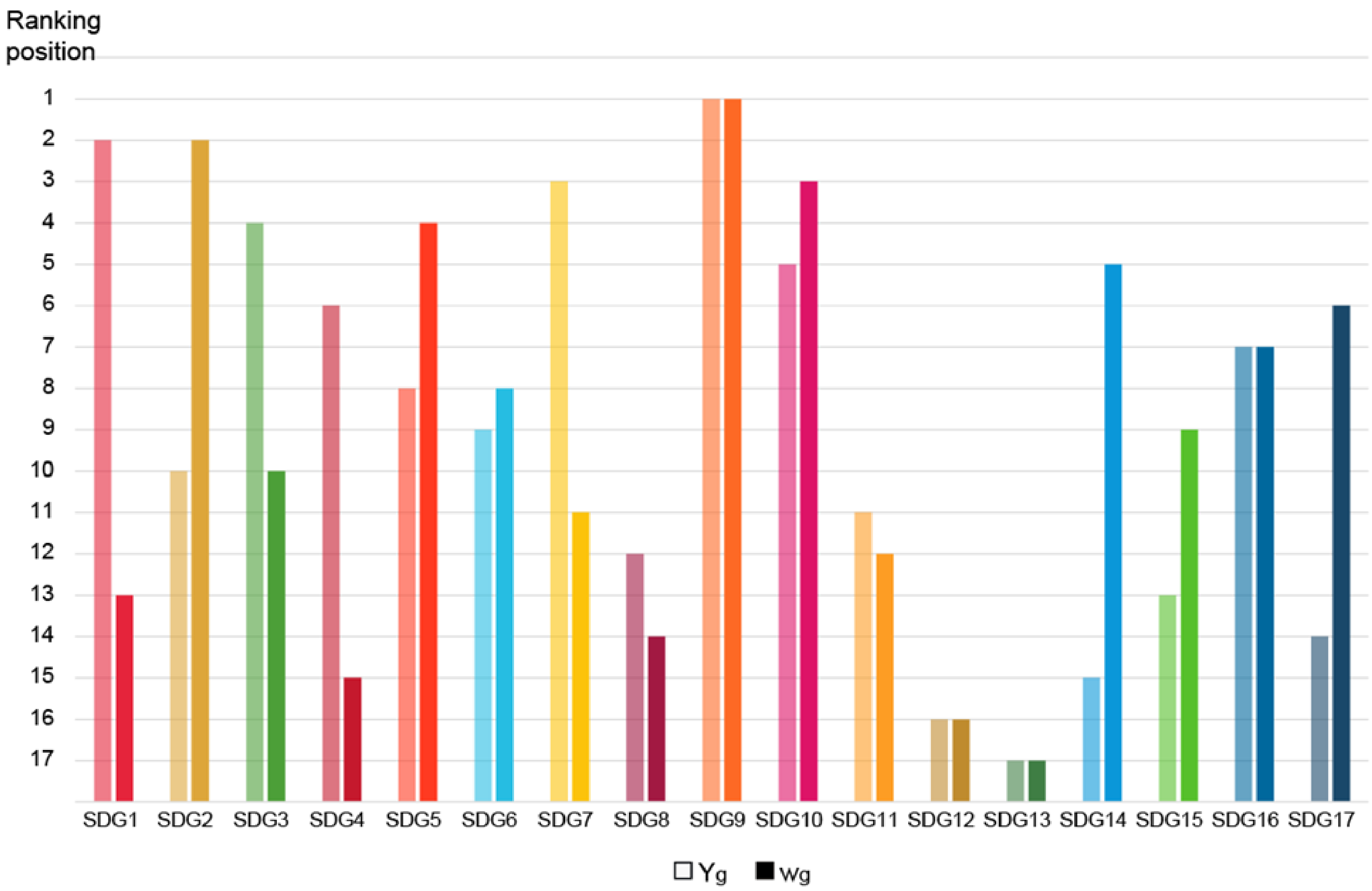

Figure S7: Bar plots of the rankings obtained from the values $\boldsymbol{Y_g}$, in transparent colours on the left, and the $\boldsymbol{w_g = Y_g/k'_g}$ ones in full colours on the right (see Eqs. (4) in the main text). From 1 to 17, Goals are sorted in descending order according to the two values.
